# Mpox-related stigma and healthcare-seeking behavior among men who have sex with men

**DOI:** 10.1186/s41256-025-00418-w

**Published:** 2025-04-14

**Authors:** Yujie Liu, Jiechen Zhang, Yong Cai

**Affiliations:** 1 Public Health Research Center, Tongren Hospital, Shanghai Jiao Tong University School of Medicine, Shanghai, 200335 China; 2 Shanghai Jiao Tong University School of Public Health, Shanghai, 200025 China; 3 Dermatology Department, Tongren Hospital, Shanghai Jiao Tong University School of Medicine, Shanghai, 200335 China

**Keywords:** Mpox, Stigma, Health-seeking behavior, Men who have sex with men

## Abstract

The 2022 global mpox outbreak highlighted significant public health challenges, with men who have sex with men (MSM) accounting for 86.7% of confirmed cases. As a high-risk group, MSM faced not only the direct health impacts of mpox but also an additional burden of stigma and discrimination, which severely hindered their willingness to seek care and access timely medical services. This article explores mpox-related stigma and discrimination and their profound impact on healthcare-seeking behaviors among MSM, drawing on evidence from global studies. We examine how stigma affects individual decision-making and has broader public health implications by exacerbating healthcare delays during the outbreak. In response, we propose actionable strategies to mitigate stigma, including providing accurate and responsible communication, strengthening community and social support network, building capacity for frontline workers, and engaging affected individuals for effective intervention. By integrating stigma-reduction measures into pandemic preparedness and response, public health systems can better support vulnerable populations, improve healthcare access, and ensure a more effective response to future outbreaks.

## Background

Mpox, a zoonotic disease caused by the monkeypox virus, was first identified in humans in the 1970s in the Democratic Republic of the Congo. For decades, the disease remained largely confined to central and western Africa. However, in 2022, the emergence of a new strain (Clade II B.1) led to increased human-to-human transmission, resulting in a rapid rise in cases across non-endemic countries. This global outbreak raised widespread concern, prompting the World Health Organization (WHO) to declare Mpox a Public Health Emergency of International Concern on July 23, 2022. As of November 30, 2024, a total of 117,663 confirmed cases and 263 deaths have been reported across 127 countries [1].

Mpox primarily spreads through close contact, including skin-to-skin, mouth-to-mouth, or respiratory exposure to infectious particles from an infected person. Epidemiological studies indicate that men who have sex with men (MSM) are disproportionately affected by mpox, accounting for 86.7% of confirmed cases [1]. Sexual contact is widely recognized as the primary transmission mode, with significant risk factors including sexual risk behavior, HIV infection, and a history of other sexually transmitted infections. These characteristics contribute to heightened stigma and discrimination toward MSM in the context of the mpox outbreak. This article aims to examine mpox-related stigma and discrimination and their impact on healthcare-seeking behaviors among MSM, while also proposing strategies to mitigate stigma and enhance healthcare access.

## Mpox-related stigma and discrimination

Health-related stigma and discrimination arise from negative attitudes and perceptions toward individuals with specific diseases or health conditions. According to the Health Stigma and Discrimination Framework [2], the process of stigmatization is driven by multiple factors at various levels, including individual beliefs and fears, interpersonal interactions, organizational and community norms, and broader public policy. These factors contribute to stigma in two distinct ways: stigma experiences refer to the direct or indirect consequences for individuals, including experienced stigma and discrimination, internalized, perceived, anticipated, and secondary stigma, while stigma practices encompass the behaviors and attitudes that perpetuate stigma, including stereotypes, prejudice, stigmatizing actions, and discriminatory attitudes. Moreover, health-related stigma often intersects with other forms of social stigma—such as those related to race, socioeconomic status, and gender identity—that are shaped and reinforced by broader systems of marginalization and discrimination [2]. For example, in societies where same-sex relationships are criminalized, MSM may avoid seeking healthcare due to fears of legal consequences. Additionally, deeply ingrained societal attitudes rooted in homophobia, classism, and racism can further exacerbate stigma against MSM.

Infectious diseases are among the most commonly stigmatized health conditions due to associations with traits such as contagiousness, danger, or incurability. The stigmatization of individuals with mpox or those in high-risk groups is similar to the early response to HIV/AIDS, which was initially labeled a “gay disease”. During the 2022 global mpox outbreak, a survey examining stigma experiences among individuals diagnosed with the disease found that respondents endorsed 4 to 16 out of 24 stigma-related items [3]. The response rates for specific stigma dimensions were as follows: 29% for personalized stigma, 13% for negative self-image, and 45% for concerns about public attitudes and disclosure [3]. The stigma and discrimination can contribute to social isolation, internalized shame, and emotional distress, significantly undermining the mental health and quality of life of affected individuals.

## Stigma, discrimination, and healthcare-seeking behavior

Stigma and discrimination have detrimental effects not only on individuals but also on public health. They create barriers that prevent those infected from disclosing their condition, accessing timely medical care, and adhering to isolation or other preventive measures, thus undermining public health efforts to control outbreaks effectively. Previous research has indicated that stigma at both individual and community levels negatively affects healthcare-seeking behavior [4]. This challenge is particularly pronounced among MSM, who often experience stigma and discrimination in healthcare settings.

Global recommendations suggest that individuals with mpox symptoms should seek medical care and self-isolate until recovery to reduce transmission risks. However, stigma have significantly impacted healthcare-seeking behaviors for mpox, resulting in lower healthcare utilization, particularly during the early stages of the outbreak. A survey conducted in the UK in June and July 2022, targeting the most affected communities (primarily MSM), found that only 29% of the participants were willing to seek medical care if they exhibited mpox symptoms [5]. Stigma deters healthcare-seeking behavior for mpox, which can contribute to an underreporting of actual cases, especially in societies where same-sex relationships are socially stigmatized. This underreporting leads to biased case estimates, which can misinform public health responses, delay resource allocation, and impede effective containment efforts.

Intersecting stigma further exacerbates these barriers by compounding discrimination resulting from the overlap of multiple identities. Research among sexual minorities has shown that individuals facing multiple forms of stigma often experience increased barriers in accessing healthcare and reduced trust in medical institutions [6]. For example, HIV-positive MSM may avoid healthcare settings due to concerns about dual stigmatization related to both HIV and mpox. Additionally, racial and ethnic minority MSM may encounter systemic discrimination in medical environments, further deterring them from seeking timely care. Understanding these intersecting stigmas is crucial for developing targeted interventions that ensure equitable access to healthcare and encourage medical engagement among at-risk populations.

## Addressing mpox-related stigma

Addressing mpox-related stigma requires a comprehensive, multi-level approach that focuses on preventing stigma before it manifests, particularly by addressing stigma drivers and modifying the norms and policies that perpetuate stigmatization [2]. Prior research highlights that effective stigma-reduction strategies must operate across multiple levels [7]. It is crucial to support individuals in coping with their experiences of stigma while simultaneously enhancing community-level activities and advocating for policy changes. We propose the following approaches to address mpox-related stigma.

### Ensuring accurate and responsible communication

Fears and misconceptions about disease transmission are an important source of stigma of infectious diseases. Therefore, timely dissemination of accurate information about mpox transmission, treatment, and prevention is essential in addressing and mitigating stigma. Governments and public health authorities should take the lead in disseminating up-to-date, evidence-based information through various channels, such as official websites and televised public service announcements, to ensure wide public reach. Additionally, distributing accessible brochures and educational videos in community centers, hospitals, and other public spaces can help enhance awareness and accessibility.

Given the rapid spread of misinformation on social media, it is crucial to regulate digital platforms to promote accurate content about mpox [8]. Social media platforms should implement robust content moderation mechanisms using technology, such as artificial intelligence and data analytics, to detect and remove misinformation in real time while actively promoting scientifically accurate information. Although digital platforms offer significant advantages in information dissemination, they should be complemented by traditional communication channels to prevent digital exclusion and ensure broader accessibility [8]. It is equally essential to establish clear media guidelines specifically for traditional outlets, outlining the use of nonjudgmental and inclusive language in reporting mpox.

### Strengthening community and social support networks

Community plays a crucial role in reducing stigma and promoting healthcare-seeking behavior. Research among people living with HIV has shown that community-driven initiatives can effectively address stigma while enhancing social support [7]. For example, the “treatment buddy” system, in which close friends or family members provide emotional support and medication reminders, has been shown to improve healthcare engagement. Similar approaches could be adapted for mpox, fostering supportive relationships to mitigate stigma at both the community and interpersonal levels. Furthermore, individuals with intersecting identities may encounter additional barriers to healthcare access and social support. Community programs can help address these challenges by establishing dedicated support groups that offer tailored resources, ensuring equitable support for all affected individuals.

### Building capacity for frontline workers

Frontline workers, in particular healthcare providers directly involved in the healthcare of mpox cases, should prioritize efforts to reduce stigma and discrimination during patient interactions. A study among MSM in Rwanda emphasizes that discriminatory behaviors, demeaning comments, and privacy violations by healthcare providers can exacerbate stigma and significantly affect healthcare-seeking behavior [9]. Therefore, comprehensive training in psychosocial support, effective interpersonal communication, and cultural sensitivity is essential to ensure that patients are treated with empathy and respect. Specifically, training should address the intersecting stigma experienced by HIV-positive and racial minority individuals, equipping providers with the knowledge and skills to deliver equitable care. To accommodate the demanding workloads of frontline workers, medical institutions can conduct regular short-term training sessions. Additionally, providing easily accessible resources, such as quick-reference guides and decision-support tools, can further assist frontline workers in promptly recognizing and addressing patients’ experience of stigmatization.

### Engaging affected individuals for effective intervention

For sustainable interventions to effectively address stigma, it is essential to involve affected individuals in the design, implementation, and evaluation of interventions [7]. During the mpox outbreak, researchers conducted interviews with individuals seeking vaccination, revealing the stigma they faced and their urgent need for public education on mpox transmission and prevention, as well as more accessible community support [10]. These findings provide valuable insights for developing more targeted interventions to reduce the burden of stigma. When engaging affected individuals, it is crucial to tailor approaches to specific contexts and subpopulations. Considering intersecting identities, such as race, socioeconomic status, and HIV status, helps address diverse experiences of stigma, ensuring that interventions are responsive to the varied needs within the MSM community.

## Implications for future public health crises

During the mpox outbreak, the impact of stigma on healthcare-seeking behaviors among MSM provides valuable insights for future public health crises. The stigma surrounding mpox underscores the importance of global health strategies that not only focus on disease control but also anticipate and mitigate stigma from the outset, preventing similar challenges in future outbreaks. For example, the Health Stigma and Discrimination Framework can be applied to develop targeted pandemic response strategies, identifying potential factors that drive or facilitate stigma across multiple levels [2]. Furthermore, reduce stigma and create supportive environments must involve clearly defined roles for government, healthcare, and community sectors. Cross-sector collaboration is essential to develop integrated, culturally sensitive interventions that address both the medical and social challenges faced by vulnerable populations.

## Conclusions

The global epidemiology of mpox underscores the critical need to recognize MSM as a high-risk group facing disproportionate challenges due to stigma and discrimination. Stigma severely impacted healthcare-seeking behaviors, particularly in the early stages of the mpox outbreak, complicating public health responses. To address this, targeted measures such as providing accurate and responsible communication, strengthening community and social support network, building capacity for frontline workers, and engaging affected individuals for effective intervention are essential. The lessons drawn from the mpox outbreak emphasize the need to integrate stigma-reduction strategies into pandemic preparedness and response frameworks. Ultimately, a coordinated, multi-sector approach is vital for combating stigma and ensuring equitable healthcare access for vulnerable populations.

## Data Availability

Not applicable.

## Abbreviations

MSM: Men who have sex with men
WHO: World Health Organization
